# Generalized logical model based on network topology to capture the dynamical trends of cellular signaling pathways

**DOI:** 10.1186/s12918-015-0249-9

**Published:** 2016-01-11

**Authors:** Fan Zhang, Haoting Chen, Li Na Zhao, Hui Liu, Teresa M. Przytycka, Jie Zheng

**Affiliations:** Biomedical Informatics Graduate Lab, School of Computer Engineering, Nanyang Technological University, Singapore 639798, Singapore; Department of Industrial Engineering and Operations Research, Columbia University, New York, NY 10027 USA; Bioinformatics Institute, Agency for Science, Technology and Research, Singapore 138671, Singapore; Lab of Information Management, Changzhou University, Changzhou, Jiangsu 213164, China; National Center for Biotechnology Information, NLM/NIH, Bethesda, MD 20894 USA; Complexity Institute, Nanyang Technological University, Singapore 637723, Singapore; Genome Institute of Singapore, Agency for Science, Technology and Research, Singapore 138672, Singapore

**Keywords:** Generalized logical model, Signaling pathways, Dynamical system, Cancer

## Abstract

**Background:**

Cellular responses to extracellular perturbations require signaling pathways to capture and transmit the signals. However, the underlying molecular mechanisms of signal transduction are not yet fully understood, thus detailed and comprehensive models may not be available for all the signaling pathways. In particular, insufficient knowledge of parameters, which is a long-standing hindrance for quantitative kinetic modeling necessitates the use of parameter-free methods for modeling and simulation to capture dynamic properties of signaling pathways.

**Results:**

We present a computational model that is able to simulate the graded responses to degradations, the sigmoidal biological relationships between signaling molecules and the effects of scheduled perturbations to the cells. The simulation results are validated using experimental data of protein phosphorylation, demonstrating that the proposed model is capable of capturing the main trend of protein activities during the process of signal transduction. Compared with existing simulators, our model has better performance on predicting the state transitions of signaling networks.

**Conclusion:**

The proposed simulation tool provides a valuable resource for modeling cellular signaling pathways using a knowledge-based method.

## Introduction

Signal transduction plays an essential role in the cellular processes in which cell responds to extracellular perturbations (e.g., the exposure to drugs or ligands). According to the signals, the cell adjust its metabolism, shape, gene expression, etc., to adapt to the environment. It is widely believed that the dysregulation of signal transduction is one of the most important pathogeneses of many human diseases including cancer. Although high-throughput experimental data show a great potential for uncovering unprecedented details of biological systems, it is still challenging to understand signaling networks at systems level. Therefore, computational simulation, which is a systems biology approach, is highly desirable for the analysis of the underlying mechanisms of how the signals are transmitted through signaling pathways.

Many existing models are able to simulate the process of signal transduction, such as Boolean network models, fuzzy logic models and kinetic models based on ordinary differential equations (ODEs). Boolean network is a simple and promising framework for the modeling of protein-protein interactions and signaling pathways. It has been used with some success in identifying stable states of a system [1, 2], simulating the influence of deletion/knockout of important nodes in a network [3], predicting carcinogenesis and targeted therapy outcomes [4], reproducing the dynamics of the yeast MAPK pathways [5], modeling the mammalian cell cycle [6] and analyzing the behaviors of the apoptosis pathways [7, 8]. However, its inability of encoding graded responses and the typically sigmoidal biological relationships becomes a significant limitation since it is able to handle only binary values, i.e., a simple on/off state which is over–simplified compared with a real signaling network. To overcome this limitation, fuzzy logic models, which generalize the on/off characteristic to a continuous range from 0 to 1, have been successfully applied to analyzing the crosstalk among the TNF/EGF/Insulin-induced signaling pathways [9] and the liver cell responses to inflammatory stimuli [10]. However, a large amount of prior knowledge is needed for the assembly of the membership functions and logical rules for the fuzzy logic models. On the other hand, ODEs have also been applied to modeling various biological processes, such as the simulation of physiological responses of mammalian cells to the control of cell cycle [11], mathematical modeling of the mechanisms for regulating the differentiation of hematopoietic stem cells [12], discovery of signaling pathway rewiring [13] and exploring the dynamics of the pathways controlling cell apoptosis [14, 15]. However, ODEs-based models require a relatively detailed knowledge of kinetic parameters which is hardly available for all the pathways. Previously, we proposed a simulation tool called SimBoolNet [16] which is based on an extended Boolean network model. Although the performance of SimBoolNet in predicting protein activities was promising [17], it has limited capability of dealing with blocking effects, degradations and sequenced perturbations.

Here, we present a generalized logical model, which is capable of revealing the process of degradation, the sigmoidal biological relationships between molecules and the effects of scheduled perturbations to signaling networks. Compared with SimBoolNet [16] and GINsim [1] (a Boolean network based simulation tool), the proposed simulator can not only predict the stable states of the signal transduction system but also dynamically simulate the effects induced by various timing and ordering of perturbations. The simulations are validated using experimental phosphoproteomics data of breast cancer cells perturbed by different combinations of drug additions [18]. The simulated time-series data of protein activity levels show significant correlations with the real time-course data, thereby demonstrating that the proposed model is able to capture the key features of the signaling pathways.

## Methods

### Computational model for dynamical simulation

**Fig. 1:**
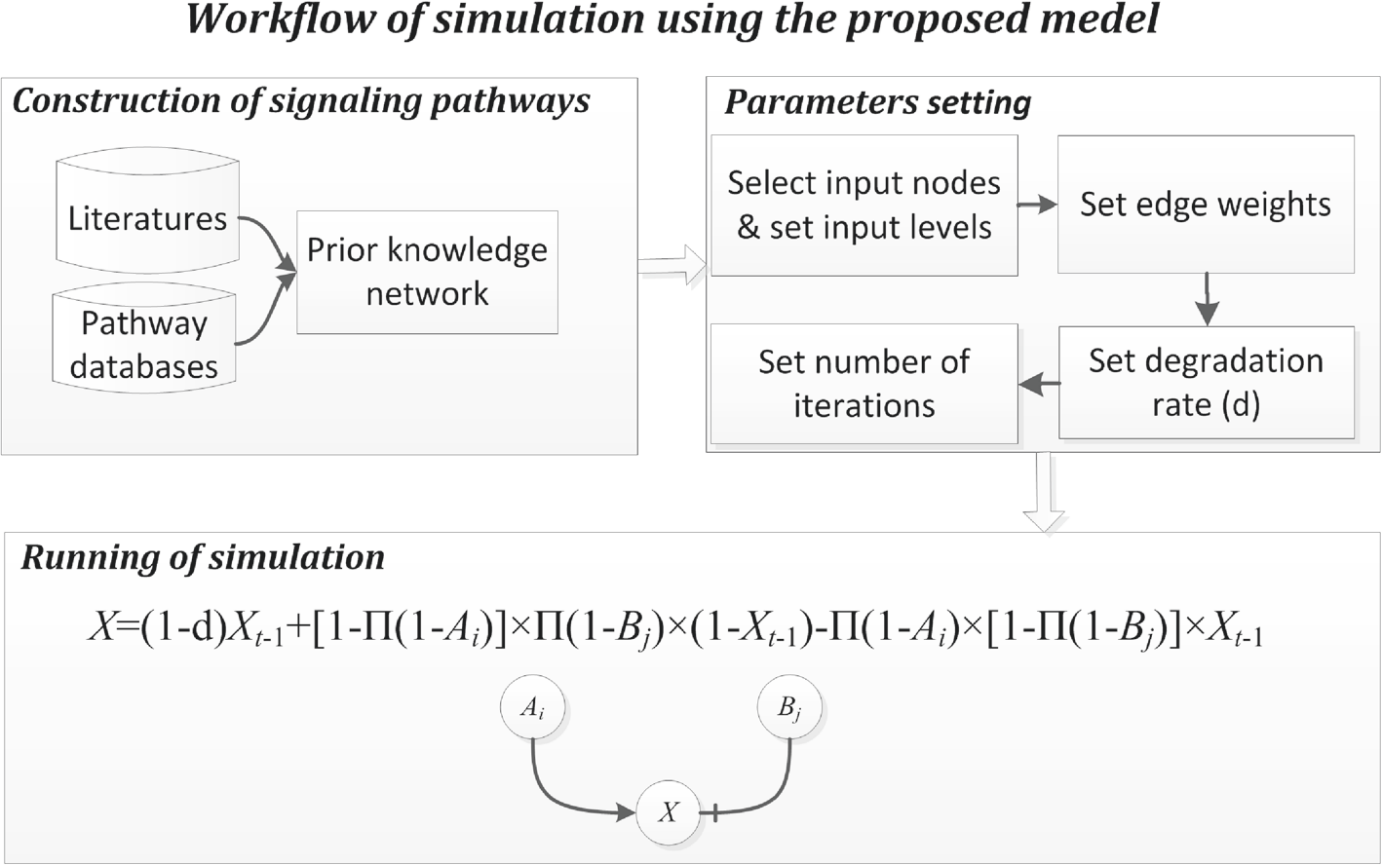
The workflow of simulation using the proposed model. The activity level of *X* is calculated based on its own previous activity with a degradation rate, and the activation and inhibition effects produced by the signals transmitted from its parent nodes

Our model takes a directed graph as the input network to do simulation. In the network, each node denotes a molecular species (e.g., a protein) and each directed edge (*u*, *v*) represents signal transduction from node *u* to node *v*. One variable with a nonnegative value from 0 (fully inhibited) to 1 (fully activated) is associated with each node to represent the activity level of the protein. The edge weight is also a variable with the value between 0 and 1 to denote the strength of the interaction and a sign (‘+’ or ‘–’) to denote the type of the interaction (i.e., positive means activation and negative blockage). Users can select input nodes and a virtual node is added upstream of all the input nodes. This virtual node is the abstraction of the extracellular environment which is able to generate signals that can stimulate or inhibit the input nodes (e.g., the receptors) of the signaling network. Given random initial activities for all the nodes in the network, their states will be updated synchronously based on their own previous states and the incoming signals from their parent nodes, according to Eq. (1). In this formula, *X*_*t*_ is the activity level of node *x* at time *t*, *d* is a pre-defined parameter denoting the degradation rate of the activated *x* from time *t*−1 to *t*, *A*_*i*_ (or *B*_*j*_) is the signals (i.e., the activity level times the edge weight) transmitted from the *i*th activating (or *j*th inhibiting) parent node upstream of *x*; $\left [1-\prod (1-A_{i})\right ]$ is the overall activating effect generated by all the incoming activating signals and $\prod (1-B_{j})$ is the probability that the incoming inhibiting signals do not affect *x*. Altogether, they act on the inactivated form of *x* at the (*t*−1)th iteration (i.e., (1−*X*_*t*−1_)). The blocking effect acting on the activated form of *x* follows the similar logic. Overall, the updated state is defined by the nondegraded part and the newly activated part minus the inhibited part. Given a user-defined number of simulation iterations, the discrete steps are employed to approximate the process that the activity levels of the nodes change over time. Figure 1 shows the workflow of the simulation using the proposed model.

(1) $$ \begin{aligned} X_{t} &= (1 - d)\times X_{t-1}+\left[1-\prod(1-A_{i})\right]\times\prod(1-B_{j}) \\ &\quad\times(1\,-\,X_{t-1}) \,-\,\prod(1\,-\,A_{i})\!\times\!\left[1-\prod(1-B_{j})\right]\times X_{t-1} \end{aligned}   $$

It is suggested that cells respond to external perturbations through a time–dependent (e.g., the schedule and duration of drug addition) process [18]. Wet–lab experiments have shown that different ordering and timing of drug additions have significantly different drug effects, such as inducing specific alterations of signaling pathways [10, 19, 20] and showing different efficiencies in killing cancer cells [18]. However, most existing simulation tools are not able to accommodate the time-staggered design of drug treatments in biological experiments. Therefore, our model introduces time-staggered perturbations to explore the effects of not only dosage, but also the schedule and duration of the perturbations to cellular systems with a knowledge-based model. The timing and the order of drug additions can be specified by users as parameters. For example, the drug can start to affect its target at the *k*th simulation iteration with a user-defined *k*. The target, input level, type of interaction (stimulation or inhibition) and schedule of the perturbations can all be specified according to user’s design of experiment.

### Network structure

**Fig. 2:**
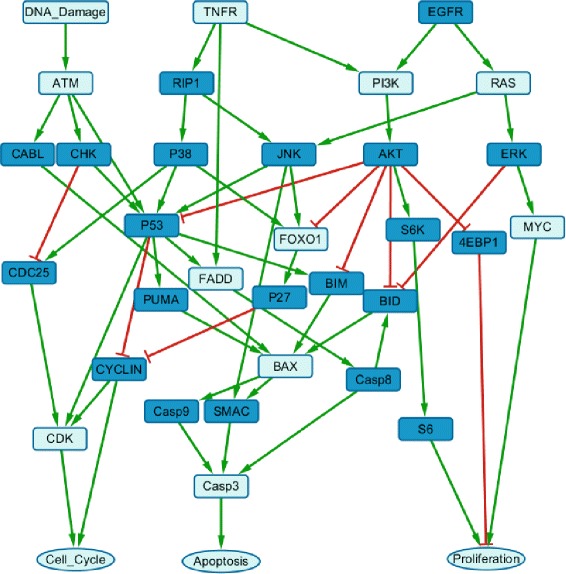
Signaling pathways constructed based on the dataset in [18]. Round rectangles and ellipses represent signaling proteins (or stimuli) and cell fates, respectively. The signals that have measurements in the dataset [18] are represented by dark blue nodes. Each activation interaction is denoted as a green edge with an arrow head and each inhibition interaction is represented by a red edge with a flat-head

A signaling network [21] (Fig. 2) is constructed according to well-known pathway databases (GeneGO MetaCore [22] and KEGG [23]). The network comprises 35 nodes, indicating 32 signaling proteins or stimuli and 3 cell fates, and 57 edges denoting signal transduction from the source nodes to the target nodes. Dark blue nodes in the pathways represent 21 signaling proteins that have been experimentally measured in [18]. Activation and inhibition interactions are denoted by green arrow and red flat-head edges, respectively.

## Results

### Performance comparison on simulating signaling responses to perturbations

For comparison, we run simulations using our program based on SimBoolNet [16], GINsim [1] and the proposed model on the same network in Fig. 2. Two different inputs are introduced: (1) the input levels of EGFR and TNFR are set to 0.5 and 0.8, respectively, (2) EGFR inhibitor is added at the 10th iteration of simulation and TNFR is activated with input level 0.8 at the 20th iteration. The number of simulation iterations is set to 100. For the proposed model, the full inhibition is denoted as -1 and the perturbations can be executed at any iteration during the simulation. The degradation rate *d* is set to 0.2. For SimBoolNet and GINsim, the blocking effect is represented by setting the activity level of EGFR to 0 from the very first step of simulation which, to our understand, is unlikely to be a precise representation. There should be a process for the inhibitor to reduce the activity level of its target, especially when the inhibitor is not added at the beginning. The edge weights of activation and blockage are set to 0.7 and 0.8, respectively. GINsim simulation, on the other hand, does not accept parameters for edge weights and the number of iterations, and executes synchronously until the system reaches the stable state. GINsim also supports the asynchronous mode, but it is a time-consuming task due to a much larger search space than with the synchronous mode. We did not get any result from running GINsim in asynchronous mode on our network (Fig. 2) within endurable time using a desktop PC (Dell Precision T3600 workstation with Intel Xeon CPU E5–1620, 8 GB RAM and Windows 7 Professional 64–bit operating system). We have also tried other different settings of input level, edge weight and degradation rate, and the results are shown in section “Model comparison and validation with real data”.

**Fig. 3:**
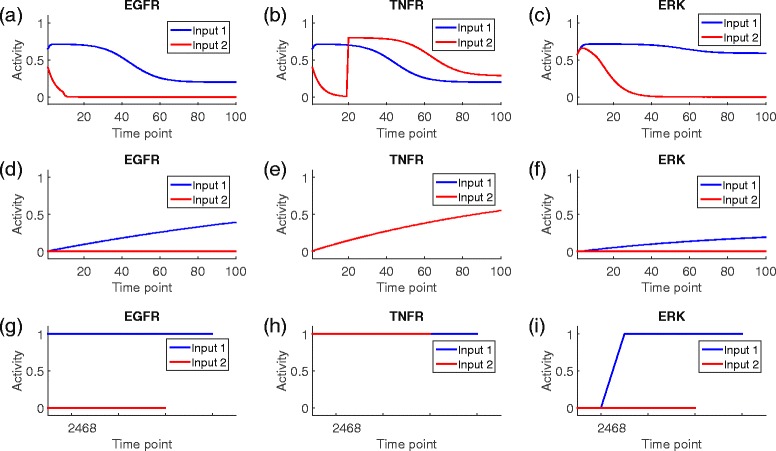
Comparison of simulation-based predictions made by using SimBoolNet, GINsim and the proposed simulator. Two different inputs are used: (1) EGFR and TNFR are perturbed at the beginning of the simulation with input levels 0.5 and 0.8, respectively, and (2) EGFR is inhibited at the 10th iteration with input level -1 and TNFR is activated at the 20th iteration with input level 0.8. The edge weights of activation and blockage are set to 0.7 and 0.8, respectively, for both inputs. The simulation is executed for 100 iterations. **a**–**c** The plots of simulation results using the proposed model for EGFR, TNFR and ERK, under two different inputs. **d**–**f** The plots of SimBoolNet results under input 1 and 2, respectively. Please note that the two curves are totally overlap for TNFR in (**e**). **g**–**i** GINsim simulation results for EGFR, TNFR and ERK. Each run of GINsim simulation executes 8 iterations before reaching the stable state

Figure 3a, b and c show the simulation results of three proteins (i.e., EGFR, TNFR and ERK) using the proposed model under the two different inputs. It can be seen that the trends of the activities of the input nodes follow an approximately sigmoidal function (the blue curves in Fig. 3a and b). When the EGFR inhibitor is added at the 10th step, the EGFR activity drops sharply within a few steps (the red curve in Fig. 3a). Consequently, the activity of its downstream node ERK (the red curve in Fig. 3c) decreases with some time delay because it takes some time for signals to be transmitted from EGFR to ERK. Under input 2, the activity of TNFR first decreases from a random initial value with a degradation rate (here is 0.2) and increases to the maximum (the input level 0.8) almost immediately at the 20th iteration (the red curve in Fig. 3b). In contrast, SimBoolNet has a limited capability of dealing with blocking effect, degradation or scheduled perturbations. We can see from the blue curves in Fig. 3d, e and f (which are outputs of SimBoolNet) that the activity levels increase monotonically from 0 to a maximum, considered as the stable state, which is unlikely to be precise in the biochemical reactions. Moreover, the inhibiting effect in SimBoolNet is represented by keeping the activity level of the target node to 0, which is unlikely to be realistic especially when the inhibiting effect should be produced in the middle of the simulation (red curves in Fig. 3d and f). Given the initial states, GINsim is able to identify the stable states [2]. However, it has similar limitations for dealing with scheduled drug additions and degradations (Fig. 3g to i).

We then compared the computational time of SimBoolNet, GINsim (in asynchronous mode) and the proposed model. In addition to the network shown in Fig. 2, a small network with 5 nodes and a large network with 500 nodes were constructed. The edges of these two networks were randomly generated. Totally, the numbers of the nodes/edges of the three networks are 5/13, 35/57 and 500/6124, respectively. The hardware being employed was Dell Precision T3600 workstation with Intel Xeon CPU E5–1620, 8 GB RAM and Windows 7 Professional 64–bit operating system. Table 1 shows the computational time of SimBoolNet, GINsim and our model on the three networks. It can be seen that the simulation with our model is faster than SimBoolNet, whereas slightly slower than GINsim when the network is small. However, GINsim has its limitation for large networks. The simulation using GINsim (in synchronous mode) on the third network with 500 nodes did not produce any result within endurable time.

**Table 1:** Comparison of the computational time required for SimBoolNet, GINsim and the proposed model

Number of nodes/edges	SimBoolNet	GINsim	Our model
5/13	9.51s	< 0.1s	0.09s
35/57	10.73s	< 0.1s	0.43s
500/6124	72.88s	Not applicable	10.68s

Three networks are employed for simulation, where the numbers of the nodes/edges are 5/13, 35/57 and 500/6124, respectively. The simulation using GINsim on the third network could not finish within endurable time (hence marked “Not applicable”)

**Fig. 4:**
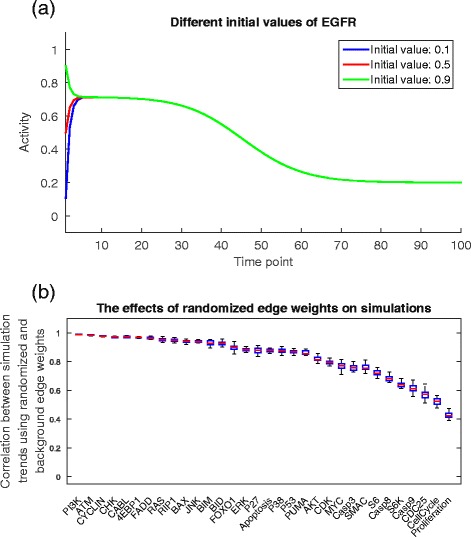
**a** The influence of different initial values on simulations. The initial value does not change the main trend of the state transition and the level of the stable state. **b** The influence of different edge weights to simulations. Each of the 32 signals shows a small range of the correlations between the simulated and the background trends, indicating that the proposed model is robust to different edge weights

The influence of different initial values on the simulation results was explored. Figure 4a shows the simulation curves of EGFR under the aforementioned input 1. Three different initial values of EGFR, namely 0.1, 0.5 and 0.9, were selected. Conclusions can be drawn from the plot that the initial value affects the time spent in reaching the stable state, but the overall trends of the state transitions and the levels of the stable states remain stable.

We went on to explore the robustness of the proposed model to the variations of edge weights. In principle, we randomly generated the edge weights to run the simulations, and then checked if the activity trend of each protein remained unchanged. For a specific input (i.e., the input levels of EGFR and TNFR are both 0.5), we first randomized the weights of all the 57 edges and ran the simulation for 100 times as the background group. For each protein, the mean activity at each time point was regarded as the background trend over time. Next, we further generated 50 groups of simulations, each group consisting of 100 simulations of randomly generated edge weights. For each group, the mean activity trend of each protein was used to calculate the correlation with the background trend. Figure 4b gives the distribution of the 50 correlations between the simulated and the background trends for the 32 non-receptor nodes (ignoring the receptors EGFR, TNFR and DNA Damage because they have no incoming edges), and the proteins are ranked based on the median of the correlations. It can be seen that 21 out of 32 proteins (i.e., from PI3K to AKT) have the medians of the correlations larger than 0.8; 10 out of 32 have the medians falling into the interval 0.5 to 0.8; and only one (i.e., Proliferation) has the median which is lower than 0.5. Moreover, all the 32 signaling proteins show small ranges of the correlations between the simulated and the background trends, indicating that the proposed model is robust for capturing the dynamical trends of the signal transduction process under different settings of edge weights.

### Model comparison and validation with real data

To estimate the performance of the proposed model, the simulated results using the network in Fig. 2 are compared with a real signaling dataset [18] containing the time-series phosphoproteomics data. In the dataset, perturbations (i.e., inhibitor of EGFR or stimuli of DNA damage or both) were applied to cells of the breast cancer cell line BT20. For each perturbation, activity levels of 32 signaling proteins (21 out of 35 are included in the network in Fig. 2) were measured at 8 time points. To simulate the perturbations, we use (*i*) half activation input signals (0.5) to represent the control situation where no stimuli or inhibitor is added; (*ii*) activation input signals (+1) to represent the addition of stimuli, i.e., the targets are fully activated; and (*iii*) inhibition input signals (–1) to represent the effect of inhibitors, i.e., the activity of the targets are suppressed.

We first simulated the dynamics of signaling without any drug addition, as a control dataset. The receptors EGFR and TNFR were selected as the input nodes with input levels both equal to 0.5. The edge weights of both activation and inhibition were set to 0.8. The simulation was executed for a number of iterations that is a multiple of 8 since there are 8 time points in the real dataset (here we chose 32 because most of the nodes reach the stable states after 30 steps). We then used Spearman correlation coefficient to measure the goodness of fit between the simulated and real data to evaluate the performance of our model. Since calculating Spearman correlation requires the two vectors to have the same length, 8 time points of the simulated data (which is the same as the real data) were extracted from the simulated time-series with equal interval, e.g., the 4th, the 8th, …and the 32nd time points. Table 2 gives the Spearman correlation coefficients for the 21 measured signaling proteins. We can see that for 16 out of 21 signaling proteins the Spearman correlation coefficients are relatively high (larger than 0.6) indicating that the simulation fits well to the real data. For the proteins with relatively poor correlation coefficients, it might because of over-simplification such as missing some cell line specific interactions in the network. For example, both S6 and S6K have only one incoming edge which is unlikely in the real cellular signaling network [24].

**Table 2:** Spearman correlations between simulated and real data

	SMAC	4EBP1	p53	ERK	S6	S6K	CABL
Correlation	0.95	0.88	0.69	0.71	0.31	0.26	–0.90
	Casp9	CDC25	CHK	p27	PUMA	AKT	JNK
Correlation	–0.60	0.62	0.21	–0.90	0.92	0.79	0.43
	p38	BIM	BID	RIP1	CYCLIN	Casp8	EGFR
Correlation	0.67	0.93	0.43	0.76	0.86	0.90	0.62

**Fig. 5:**
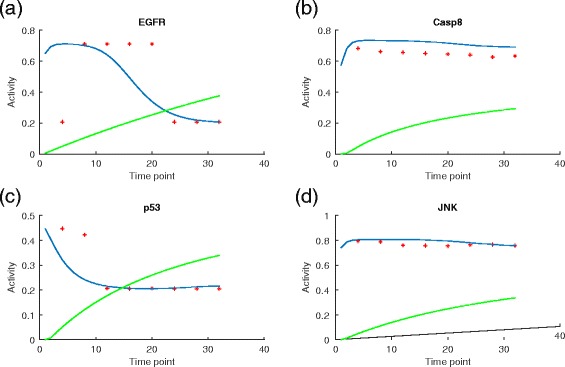
The plots of the simulated and real data in the control group. The blue and green curves are the simulated data using the proposed model and SimBoolNet, respectively. The red dots are the real data. The four panels (**a**), (**b**), (**c**) and (**d**) correspond to the plots of four proteins EGFR, Casp8, p53 and JNK, respectively

On the other hand, since the simulation is an approximately continuous process while the wet–lab experiment measured only at a few selected time points, we can scale the discrete dots in the real data to align with the 32 simulated steps by multiplying the index of each time point in the real data by 4 to match the index of each iteration in the simulation. For comparison, SimBoolNet was also employed to do the simulation under the same inputs, i.e., the input levels of EGFR and TNFR are both 0.5 and the edge weights are all 0.8. Figure 5 shows the plots of the simulated (scatter plots each with a trend line) and real data (scatter plots) for EGFR, Caspase 8, p53 and JNK. The blue and green curves represent the simulated data using the proposed model and SimBoolNet, respectively, while the red dots represent the real data (normalized to the same scale as the simulated data). In Fig. 5a, the simulation curve of EGFR captures the trend of a quick drop from a plateau to the flat bottom although there is a time delay. The slow and small decrease of the activity of Caspase 8 is also captured by our simulation as shown in Fig. 5b. For p53, the simulation shows a high decreasing rate at the beginning and the activity of p53 reaches the stable state quickly, which agrees with the trend of the real data. The gradient of the JNK simulation curve also fits the real data although the starting point of the dropping lags behind, which suggests that it is important to scale the simulated timing to accurately match the real time points. To address the issue of time scale would be an important future work. By contrast, the simulated trends of SimBoolNet are mainly monotonically increasing which cannot fit the real data well.

We then simulated the state transitions of signaling pathways under the perturbations in Table 3. For example, we gave half activating (+0.5) and full blocking (–1) signals to TNFR and EGFR, respectively, to simulate the addition of EGFR inhibitor. The edge weights of activation and inhibition were set to 0.7 and 0.8, respectively. The number of simulation iterations was set to 32, and the degradation rate 0.2.

**Table 3:** Inputs of the simulation in section “Model comparison and validation with real data”

	EGFR	TNFR	DNA damage
Control	+0.5	+0.5	random value
EGFR inhibitor	–1	+0.5	random value
DNA damage stimuli	random value	+0.5	+1
Both drugs	–1	+0.5	+1

The symbols ‘+’ and ‘–’ represent the perturbation types, i.e., activation and inhibition. The columns are the input nodes of simulation and the rows are various conditions including the treatments of biological experiments with drugs corresponding to wet-lab experiments in [18]

It has been known that the in vivo drug effect on the signaling pathways is through the change of the activities of the proteins downstream of the drug targets [20, 25]. Therefore, for these downstream proteins, if the simulation data based on the perturbed inputs (rows 2 to 4 in Table 3) fit the experimental data better than the simulations using the control input (rows 1 in Table 3), we believe our simulator captures the main influences of the drugs on the networks. For example, the second and the third columns of Table 4 show the correlation coefficients between the real data and the simulations using the control input and EGFR inhibitor input (rows 1 and 2 in Table 3), respectively. It can be seen that, for the nodes downstream of EGFR (such as AKT, S6, S6K and BIM), the fitness of simulations to the drugged data (biological measurements treated with drug which targets at EGFR) is significantly improved when the control input is replaced by the EGFR inhibitor input. A similar conclusion can be drawn for the proteins downstream of DNA damage, including p53, CABL and Caspase 8, when the simulations using the control input and DNA damage stimuli as input are employed to fit the real data (the forth and the fifth columns of Table 4). Moveover, it is believed that the treatment with both drugs have a higher efficiency on killing cancer cells than that with a single drug [18] which may be explained by the characteristics of SMAC and Caspase 8 (both upstream of Caspase 3), in that the improvement of fitness is mainly achieved under the treatment of both drugs (the last two columns of Table 4). There are proteins, such as ERK and CHK (downstream of EGFR and DNA damage, respectively), that do not follow the above patterns, probably due to our insufficient knowledge about signaling pathways.

**Table 4:** Goodness of fit of the simulations to the real experimental measurements under drug treatments

	Control	EGFR	Control	DNA damage	Control	Both
	input	inhibitor	input	stimuli	input	drugs
AKT	0.64	0.81	–0.40	–0.54	0.75	0.93
ERK	0.32	0.40	–0.30	–0.39	0.19	0.23
S6	0.53	0.86	0.42	0.57	0.52	0.89
S6K	0.49	0.84	0.35	0.66	0.58	0.96
4EBP1	0.67	0.67	–0.33	-0.33	0.92	0.92
BIM	0.32	0.72	0.35	0.65	–0.15	–0.38
BID	0.30	0.37	0.03	0.03	0.16	0.16
JNK	0.02	–0.07	–0.47	–0.58	–0.06	0.01
p53	–0.04	–0.45	0.21	0.83	0.01	–0.09
CABL	–0.45	–0.52	0.04	0.55	0.21	0.78
CHK	–0.12	–0.18	–0.25	–0.28	–0.30	–0.69
CDC25	0.29	0.23	0.15	0.33	0.18	0.64
Casp8	0.06	–0.06	0.14	0.72	0.26	0.68
SMAC	–0.05	0.08	–0.71	–0.87	0.48	0.99

For example, the second column is the correlation coefficients between the simulations using control input (row 1 in Table 3) and the biological measurements treated with the drug that targets EGFR

## Discussion and conclusion

In this paper, we present a model to dynamically simulate the process of intracellular signal transduction. According to a phosphoproteomics dataset [18], we constructed a network, which comprises 35 nodes (21 nodes have experimental measurements) and 57 edges, to do the simulation. The state of each node is calculated based on its own previous state with a degradation rate, and the activation and inhibition effects produced by the signals transmitted from its parent nodes. Different combinations of perturbations were applied to the network. The simulation results have been evaluated with the real data, demonstrating that our simulator has the ability to grasp the main dynamical trends of signal transduction. Compared with SimBoolNet [16] and GINsim [1], the proposed model shows promising performance in revealing the graded responses, the sigmoidal biological relationships and the effects of scheduled perturbations to a signaling network. Moreover, by testing the proposed model with different values of parameters (e.g., the initial activities of the proteins and the edge weights), we have shown that our method performs robustly in revealing the dynamics of the signaling pathways when the prior knowledge of the network topology is reliable.

Studying the cell responses to extracellular perturbations is a major endeavor for biomedical research and pharmaceutical industry. With the development of high-throughput experiments, large-scale data are available to help uncover important biological mechanisms at systems level. However, most existing data-driven methods [25] have limitations in revealing underlying molecular mechanisms. Therefore, computational simulation based on the integration of prior knowledge with data shows a great potential for revealing insights into the dynamical system of signal transduction, and thus would be a valuable complement to the data-driven methods. Although the proposed model is still limited in mapping the simulation steps to the experimental time points, we believe that the integration of both knowledge and data, such as learning the edge weights from experimental data, would be a powerful approach to understanding the signal transduction networks. In addition, generalization of the present model, which uses the synchronous updating scheme, such that it is able to deal with asynchronous dynamics, e.g., updating a randomly selected node at each time point, would also be a valuable future direction.
